# Growth and Brilliant Photo-Emission of Crystalline Hexagonal Column of Alq_3_ Microwires

**DOI:** 10.3390/ma11040472

**Published:** 2018-03-22

**Authors:** Seokho Kim, Do Hyoung Kim, Jinho Choi, Hojin Lee, Sun-Young Kim, Jung Woon Park, Dong Hyuk Park

**Affiliations:** 1 Department of Applied Organic Materials Engineering, Inha University, Incheon 22212, Korea; seokho@inha.edu (S.K.); gg1236@inha.edu (D.H.K.); jinho@inha.edu (J.C.); hojin@inha.edu (H.L.); 22161060@inha.edu (S.-Y.K.); jungwoon@inha.edu (J.W.P.); 2 Department of Chemical Engineering, Inha University, Incheon 22212, Korea

**Keywords:** organometal, Alq_3_, photoluminescence, crystallinity, surfactant, confocal microscope

## Abstract

We report the growth and nanoscale luminescence characteristics of 8-hydroxyquinolinato aluminum (Alq_3_) with a crystalline hexagonal column morphology. Pristine Alq_3_ nanoparticles (NPs) were prepared using a conventional reprecipitation method. Crystal hexagonal columns of Alq_3_ were grown by using a surfactant-assisted self-assembly technique as an adjunct to the aforementioned reprecipitation method. The formation and structural properties of the crystalline and non-crystalline Alq_3_ NPs were analyzed with scanning electron microscopy and X-ray diffraction. The nanoscale photoluminescence (PL) characteristics and the luminescence color of the Alq_3_ single NPs and their crystal microwires (MWs) were evaluated from color charge-coupled device images acquired using a high-resolution laser confocal microscope. In comparison with the Alq_3_ NPs, the crystalline MWs exhibited a very bright and sharp emission. This enhanced and sharp emission from the crystalline Alq_3_ single MWs originated from effective π-π stacking of the Alq_3_ molecules due to strong interactions in the crystalline structure.

## 1. Introduction

Organic semiconductors have emerged as a major class of plausible candidates for achieving low-cost, flexible and high-efficiency electronic devices [1,2]. Intensive research is currently carried out worldwide using crystalline organic semiconductors as active materials for optoelectronic devices because of the semiconducting characteristics and the excellent photo-induced charge creation of these molecules [3]. Single-crystalline organic small molecules are among the best recognized materials, as there are no grain boundaries and few charge trapping sites [4,5]. Among the various organic small molecules, organometallics have attracted much attention in recent years owing to their unique optical and electrical properties and their various applications [6,7]. Organometallic molecules are composed of a central metal surrounded by an organic conjugated structure. These combinations of metals and organic compounds generally furnish optoelectronic properties. These properties can be controlled by altering structural features such as the crystallinity and molecular aggregation, or by chemical processes (e.g., doping or substitution). One interesting phenomenon of organometallic molecules is their light-induced or electrically-driven luminescence. Many efforts have been made to improve luminous efficiency, including the use of fluorophores.

The best known of these fluorophores—8-hydroxyquinolinato aluminum (Alq_3_)—exhibits excellent optical properties that can be exploited in optoelectronic devices, such as its use as the active light emissive layer of organic light emitting diodes (OLEDs) [8,9]. Generally, Alq_3_ dissolved in an organic solvent emits bright yellow-green under ultraviolet (UV) irradiation. However, in the solid-state, amorphous Alq_3_ exhibits very low brightness under the same UV irradiation conditions due to random stacking in the molecules. Growth of this architecture for application to a variety of optoelectronic devices can thus be problematic. Recent studies have reported various methods for the growth of regular and crystalline Alq_3_ morphologies, such as hexagonal columns, using vapor deposition or solvent treatment. These approaches lead to enhanced optical properties due to enhanced crystallinity or molecular orientation [10,11].

In recent years, organic small molecule crystals have attracted increasing research interest due to their potential use in optoelectronics and photonics. Much research effort has been devoted to molecule design and crystal growth to achieve the desired function [12,13]. The electronic and optical properties of organometallics are fundamentally different from those of inorganic metals and semiconductors due to the weak intermolecular van der Waals forces. Therefore, understanding and controlling the arrangement of molecules in the solid-state are fundamental issues for obtaining the desired chemical and physical properties. These non-covalent intermolecular interactions, such as hydrogen bonding and π-π stacking, can strongly influence the final packing structure [14,15]. Moreover, there are relatively few reports on the optical characteristics of single units in the solid-state. Thus, this study focuses on the nanoscale photoluminescence (PL) characteristics of Alq_3_ crystals in the solid-state in an attempt to examine the changes in the structural and optical properties upon crystallization [16,17].

**Figure 1:**
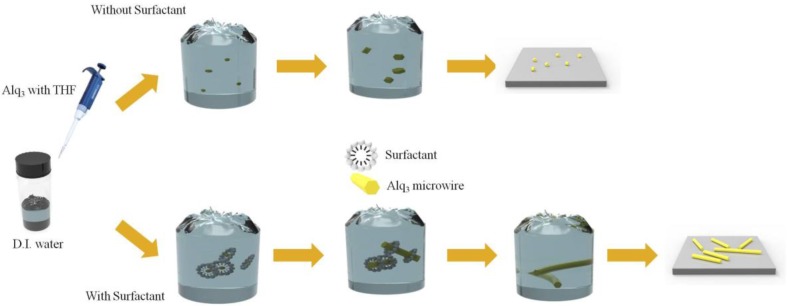
Schematic illustration of surfactant-assisted organic crystal growth using reprecipitation method.

Herein, we report the growth and nanoscale luminescence characteristics of single-crystalline Alq_3_ microwires (MWs) as shown in Figure 1. Crystalline Alq_3_ MWs with a hexagonal column arrangement can be grown via surfactant-assisted self-assembly using sodium dodecyl sulfate (SDS) [18]. The surfactant induces the formation of micelles in deionized (D.I.) water and the Alq_3_ molecules penetrate the micelle and initiate the nucleation reaction. Because organometallic molecules are not amenable to water due to their inherent polarity, they penetrate the micelle to reduce surface energy. After nucleation, the Alq_3_ molecules act as seeds for growth of the hexagonal 1D MWs. We demonstrate that hexagonal column crystal Alq_3_ has a much brighter emission profile than randomly oriented Alq_3_ nanoparticles in a single unit.

## 2. Materials and Methods

Sample preparation: Alq_3_ (C_27_H_18_AlN_3_O_3_, purity 99.995%) and SDS (CH_3_(CH_2_)_11_OSO_3_Na, purity 99.0%) were purchased from Sigma Aldrich (St. Louis, MI, USA). Alq_3_ was dissolved in tetrahydrofuran (THF) to give a concentration of 4 mg·mL^−1^. The Alq_3_ solution was heated to 50 °C and homogenized using a magnetic stirrer for complete dissolution; SDS (4 mg·mL^−1^) was prepared in D.I. water.

The SDS solution was poured into a 20-mL vial and vigorously magnetically stirred on the hot-plate. The Alq_3_ solution was added and forcefully ejected into the SDS solution using a micro-pipette. The vial was capped and stirring was continued at a high rpm for 15 min. The mixture was kept in an oven at 50 °C for 40 h. Another solution was prepared with the same formulation used above, without the addition of SDS.

Measurement: The surface morphology of the Alq_3_ MWs was analyzed using a field-emission scanning electron microscope (SEM; Hitachi, Tokyo, Japan, SU-8010) at an acceleration voltage of 15 kV. The powder X-ray diffraction (XRD; X’Pert Powder Diffractometer, PANalytical) patterns were captured at a voltage of 40 kV and a current of 40 mA using Cu-Kα radiation (λ = 1.540 Å). The scan rate was 0.02°·s^−1^ and the 2θ range was from 2° to 60°. The luminescence color charge-coupled device (CCD) images of Alq_3_ were acquired with an AVT Marlin F-033C (λ_ex_ = 435 nm) instrument. To compare the brightness (i.e., luminescence intensity) of the CCD images of the particles and crystals of Alq_3_, the irradiation time was fixed at 0.1 s. Laser confocal microscope (LCM) PL spectra were acquired with a homemade LCM instrument [19,20,21,22]. The 405 nm line of an unpolarized diode laser was used for the LCM PL excitation. The organic crystals were placed on a glass substrate, which was mounted on the xy-stage of the confocal microscope. An oil-immersion objective lens (N.A. of 1.4) was used to focus the unpolarized laser light on the crystal. The spot size of the focused laser beam on the sample was approximately 200 nm. The scattered light was collected with the same objective lens and the excitation laser light was filtered out with a long-pass edge-filter (Semrock, Rochester, NY, USA). The red-shifted PL signal was focused onto a multimode fiber (core size = 100 μm) that acted as a pinhole for the confocal detection. The other end of the multimode fiber was connected to the photomultiplier tube for acquisition of the PL image, or the input slit of a 0.3 m long monochromator equipped with a cooled charge coupled device for acquisition of the PL spectra. Solid-state PL measurements could therefore be performed at the nanometer scale. The laser power incident on the sample and the acquisition time for each LCM PL spectrum were fixed at 500 μW and 1 s, respectively, for all confocal PL measurements.

## 3. Results

### 3.1. Morphological Analysis

**Figure 2:**
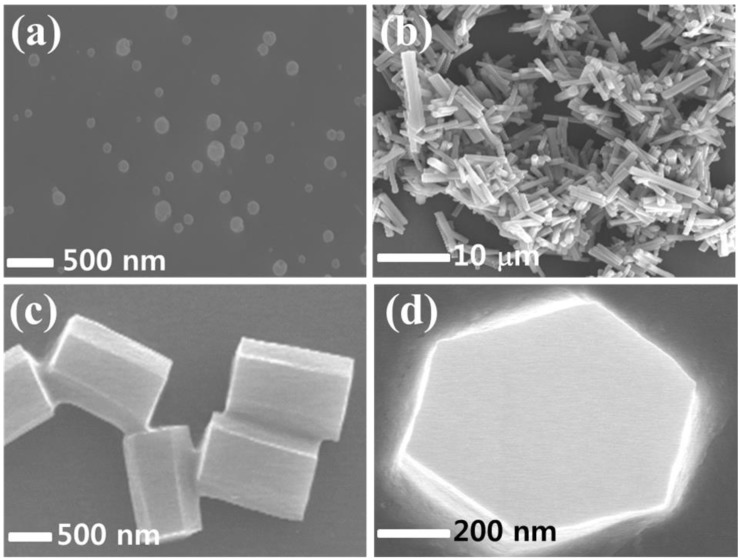
Scanning electron microscopy (SEM) images of (**a**) Alq_3_ NPs and (**b**) crystalline hexagonal columns of Alq_3_. High-magnification SEM images of (**c**) side view and (**d**) top-view of crystal hexagonal columns of Alq_3_.

The formation of the Alq_3_ NPs and the crystal hexagonal column of Alq_3_ MWs was visually observed through SEM imaging, as shown in Figure 2a,b,. The mean diameter of the Alq_3_ NPs was estimated to be 150 (±50) nm, as shown in Figure 2a. The NPs grew randomly into sphere-like plates or particles because the Alq_3_ molecules aggregated randomly in the solvent. Thus, there was no specific shape or direction. When a surfactant was employed along with the reprecipitation process, crystalline hexagonal columns were formed with growth in only one specific direction. This is because the SDS (as a surfactant) micelles assist the nucleation of Alq_3_ during reprecipitation. The Alq_3_ nuclei can act as seeds for growth of the hexagonal column. We found that the cross-section of the Alq_3_ single crystal was hexagonal, based on the tilted SEM image, with a length of 5–30 μm depending on the growth conditions, such as the time, solvent temperature and concentration—as shown in Figure 2b. These results suggest that the SDS surfactant transformed the amorphous non-crystalline Alq_3_ NPs into a crystalline phase with growth in a 1D manner, resulting in the hexagonal column MWs. The side-view and top-view high-magnification SEM images clearly confirm the formation of highly ordered crystalline hexagonal columns of Alq_3_ MWs with a mean length and diameter of 5 μm and 1 μm as shown in Figure 2c,d, respectively.

### 3.2. Optical Properties

**Figure 3:**
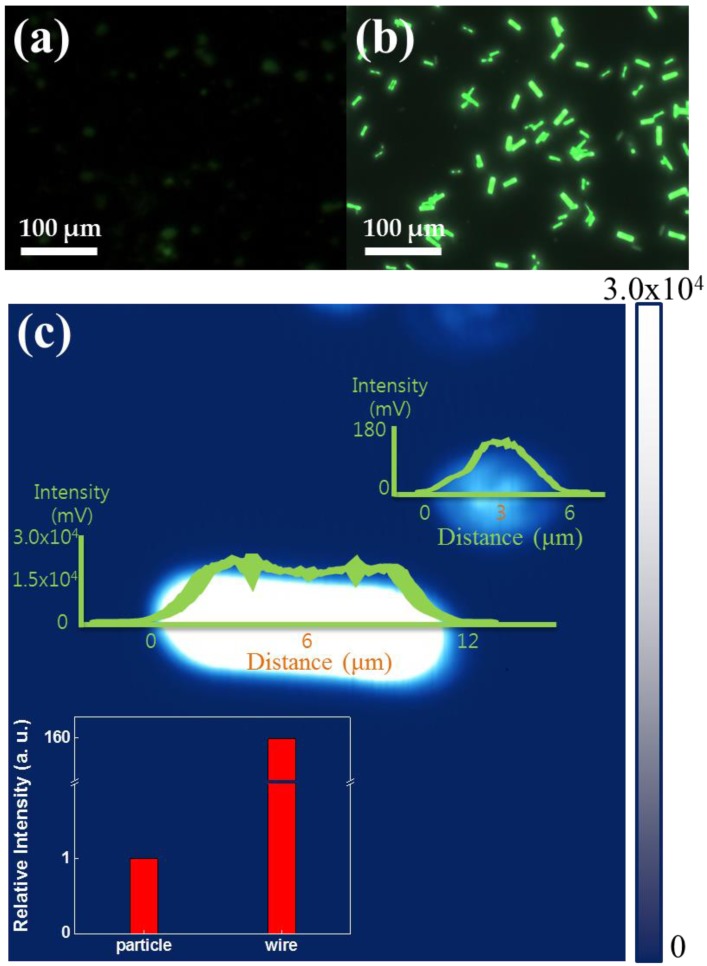
Color charge-couple device (CCD) images of (**a**) Alq_3_ NPs and (**b**) crystal hexagonal column of Alq_3_ MWs. (**c**) Photoluminescence image of both crystal Alq_3_ MW and amorphous Alq_3_ particle. (inset shows relative luminescence intensity of particle and wire).

Figure 3a,b show the CCD images used for direct observation of the luminescence color of the Alq_3_ NPs and crystal hexagonal columns of Alq_3_ MWs, respectively. The color CCD image of the crystal hexagonal column of Alq_3_ MWs showed much brighter luminescence than that of the Alq_3_ NPs. A weak green emission was observed for the Alq_3_ NPs because of their amorphous nature, as shown in Figure 3a. The crystal hexagonal column of Alq_3_ MWs emitted bright green, as shown in Figure 3b. Figure 3c shows the two-dimensional (2D) LCM PL images for an isolated Alq_3_ NP and crystal hexagonal column of Alq_3_ MW, respectively. Under identical LCM PL imaging conditions (i.e., simultaneous comparison), a much brighter emission was observed for the crystal hexagonal column of Alq_3_ MWs than for the Alq_3_ single NP. The measured voltages of the photomultiplier tube output from the z-axis representing the PL intensity of the single strand of the crystal hexagonal column of Alq_3_ MWs was 25–31 V, which is about 150–200 times higher than that (0.12–0.15 V) of the amorphous Alq_3_ single NP, as shown in Figure 3c.

**Figure 4:**
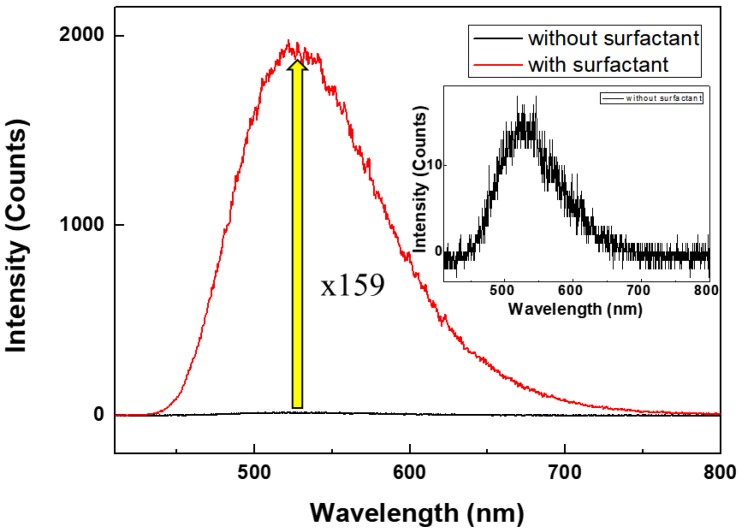
Comparison of solid-state laser confocal microscope (LCM) photoluminescence (PL) spectra of nanoscale crystal and amorphous Alq_3_ (inset shows solid-state spectrum for amorphous Alq_3_).

Figure 4 shows the variation in the PL spectra of Alq_3_ depending on its crystallinity and structure. A homemade laser confocal microscope system was used for nanoscale measurement of the PL for a single unit. Solution-state PL spectra were also acquired using a polymer cuvette. For the solvated crystalline Alq_3_, the PL intensity was 1.6 times higher than that of amorphous Alq_3_ due to auxiliary interactions between the incident light and solution, such as scattering, diffraction and reflectance. On the other hand, for nanoscale solid-state Alq_3_ immobilized on a substrate, the individual crystals were separated and there were no auxiliary interactions. For all spectra, the main peak was positioned around 530 nm. However, the LCM PL intensity for the single unit of Alq_3_ MWs was significantly higher due to the crystalline structure. The PL signal for a single unit of the crystal hexagonal column of Alq_3_ MWs was 159 times more intense than that of amorphous Alq_3_. The highly enhanced luminescence characteristics of the crystalline Alq_3_ materials originates from the strong π-π interactions of the Alq_3_ molecules, given that energy transfer is efficient in the Alq_3_ crystals and there is less defect emission.

### 3.3. Structural Properties

**Figure 5:**
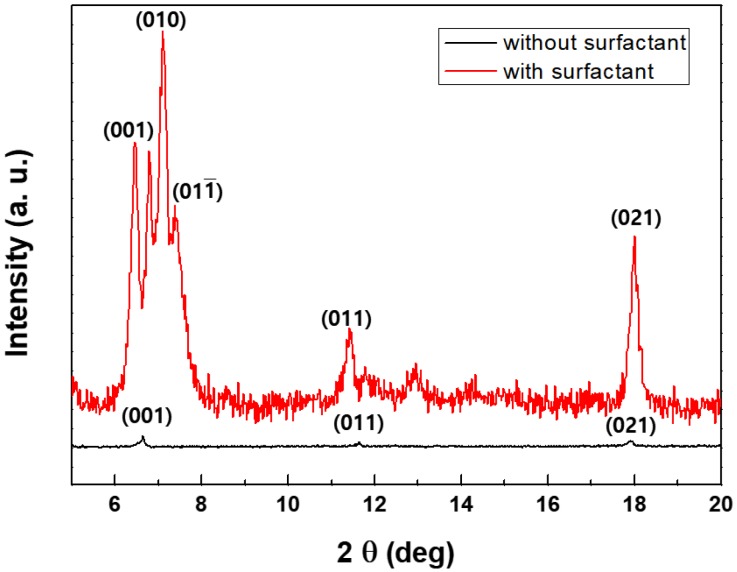
Comparison of X-Ray Diffraction (XRD) spectra of Alq_3_ (upper line corresponds to crystal hexagonal column microwires (MWs), lower line indicates amorphous nanoparticles (NPs)).

To confirm the relationship between the crystallinity and PL efficiency of Alq_3_, X-ray diffraction data were obtained for the samples prepared, with or without SDS as a surfactant. Figure 5 displays the XRD patterns for the two types of Alq_3_. The XRD peaks indicate the degree of crystallinity according to Bragg’s law. The peak corresponding to the (001) crystal lattice plane was observed at 6.47° for crystalline Alq_3_. Peaks were also observed at 11.55° and 17.45° for both cases, respectively, corresponding to the (011) and (021) planes. However, other major peaks at 7.1°, 7.38°—related to the (010) and (011) planes respectively—were only observed for the crystalline structure. We can also identify the crystal phase of Alq_3_ using XRD data. In Figure 5, the XRD pattern of Alq_3_ MRs showed 6.47°, 7.1° and 11.55° as related to typical α-phase peaks, in addition to δ-phase peaks at 6.78° and 7.38° [23,24]. The XRD results indicate that strong π-π interactions are operative along the *b*-axis (*b* = 6.47 Å) of the Alq_3_ molecules in the hexagonal column of Alq_3_ MWs, which contributes to the very bright emission [25,26]. However, relatively weak XRD peaks were observed for the Alq_3_ NPs, which have the same molar concentration as Alq_3_ MWs—as shown in Figure 5—indicating the poor crystalline structure of the Alq_3_ NPs. This led to a weak and broad PL spectrum for the Alq_3_ NPs. The Alq_3_ crystals prepared with SDS grew preferentially in a specific direction and were more crystalline than the congener prepared without SDS, based on the data shown in Figure 5.

## 4. Conclusions

Single-crystalline hexagonal columns of Alq_3_ MWs were fabricated through a surfactant-assisted reprecipitation method. The crystalline hexagonal Alq_3_ single MWs exhibited very bright emission with sharp peaks, as observed in the color CCD images and LCM PL images and spectra. The main PL peak at 530 nm for the crystalline Alq_3_ single MW had a relatively narrow full-width-at-half-maximum. The intensity of the LCM PL peak at 530 nm for the single hexagonal column of Alq_3_ MWs was approximately 160 times higher than that of the Alq_3_ NPs, representing a dramatic increase. The highly enhanced luminescence characteristics of the crystalline Alq_3_ materials originates from the strong π-π interactions of the Alq_3_ molecules along the *b*-axis in the single crystalline form. Also, the surfactant present in crystals of π-π stacking has an effect on the active enhanced fluorescence and reduces the vibrational losses of the molecular structure to achieve strong fluorescence emission.
